# Limits of Perceived Audio-Visual Spatial Coherence as Defined by Reaction Time Measurements

**DOI:** 10.3389/fnins.2019.00451

**Published:** 2019-05-22

**Authors:** Hanne Stenzel, Jon Francombe, Philip J. B. Jackson

**Affiliations:** ^1^Centre for Vision, Speech and Signal Processing, University of Surrey, Guildford, United Kingdom; ^2^BBC Research & Development, Salford, United Kingdom

**Keywords:** ventriloquism, Simon effect, spatial correspondence, reaction times, audio-visual

## Abstract

The ventriloquism effect describes the phenomenon of audio and visual signals with common features, such as a voice and a talking face merging perceptually into one percept even if they are spatially misaligned. The boundaries of the fusion of spatially misaligned stimuli are of interest for the design of multimedia products to ensure a perceptually satisfactory product. They have mainly been studied using continuous judgment scales and forced-choice measurement methods. These results vary greatly between different studies. The current experiment aims to evaluate audio-visual fusion using reaction time (RT) measurements as an indirect method of measurement to overcome these great variances. A two-alternative forced-choice (2AFC) word recognition test was designed and tested with noise and multi-talker speech background distractors. Visual signals were presented centrally and audio signals were presented between 0° and 31° audio-visual offset in azimuth. RT data were analyzed separately for the underlying Simon effect and attentional effects. In the case of the attentional effects, three models were identified but no single model could explain the observed RTs for all participants so data were grouped and analyzed accordingly. The results show that significant differences in RTs are measured from 5° to 10° onwards for the Simon effect. The attentional effect varied at the same audio-visual offset for two out of the three defined participant groups. In contrast with the prior research, these results suggest that, even for speech signals, small audio-visual offsets influence spatial integration subconsciously.

## 1. Introduction

Audio-visual spatial perception has been studied for decades. It has been shown that spatially separated signals may be perceived at the same position, the so-called ventriloquism effect. The current paper investigates the limits of this audio-visual spatial fusion using indirect reaction time (RT) measurements.

A new interest in the field arises from recent developments in consumer technology introducing immersive, 3D audio-visual playback devices. This technology aims at recreating a fully immersive, 360°, audio-visual scene in which consumers can look around and navigate. The reproduced visual signals inherently contain spatial information. A fully surrounding, interactive and responsive 360° audio spatial scene, however, requires reproduction procedures that are technically complex and computationally expensive on a number of layers. It is thus prone to errors and subject to simplification efforts. Both types of signal degradation introduce a coarser audio spatial environment and lead to spatial mismatches between presented visual and audio spatial information. Generic rules on the limits of audio-visual spatial perception, based on perceptual data, are therefore essential for quality monitoring and assurance of perceptually satisfactory technical solutions and simplifications. In order to obtain these perceptual limits, it is necessary to evaluate the audio-visual offset at which spatial misalignment starts to affect our perception noticeably.

Across literature, the reported perceivable audio-visual offset varies strongly as presented in Table 1. Differences range from just-noticeable-differences (JND) of 4° for unnatural signals (Sporer et al., 2015) to a point of subjective equality (PSE) of 19° for speech when elicited by untrained participants (Stenzel et al., 2017a). These large differences hinder the process of defining limits for qualitative monitoring of audio-visual offsets. The large variations across experiments show a dependency on numerous factors, such as participant training (Komiyama, 1989; Stenzel et al., 2017a), type of sound (Jackson, 1953; Warren et al., 1981; Stenzel and Jackson, 2018), and test setup (Lewald and Guski, 2003). These dependencies result from the direct evaluation, leading to biased results (Pike and Stenzel, 2017) as all these methods require participants to be aware of the aspect under test (Shamma et al., 2011). Indirect measures may, therefore, be more feasible in the determination of the maximally acceptable audio-visual spatial offset. Nevertheless, any kind of limit definition should be based on realistic, ecologically valid stimuli to justify their application.

**Table 1:** Summary of papers on the limit of ventriloquism in audio-visual application settings.

**Study**	**Tr**.	**Stimulus**	**Setup**	**Type of test**	**Results**
de Bruijn and Boone, 2003	X	Synchr. speech (AV)	3D video projection, WFS, loudspeakers	Absolute 5-point impairment scale	No values given
Melchior et al., 2006	X	Pink noise (A) with 3D object (V)	WFS, VR device	5-point impairment scale with hidden anchor	4°–8°
Bertelson and Aschersleben, 1998	X	2 kHz pulses (A) with LED light flashes (V)	Phase panning between two loudspeakers, central LED	Staircase paradigm, JND	~5°
Sporer et al., 2015	T, U	“Meaningless speech” (A), pink noise (A), 10 cm white dot (V)	Wall of loudspeakers, interpolated panning, video projection	Staircase paradigm, JND	4°–7°
Melchior et al., 2003	T	Synchr. speech (AV)	WFS, 2D projection	5-point impairment scale with hidden anchor	5°–7°
Komiyama, 1989	T, U	Synchr. speech (AV), Synchr. singing voice (AV)	Loudspeakers at every 5°, HDTV	Absolute 5-point impairment scale	11° (T) 20° (U)
Stenzel et al., 2017a	T,U	Synchr. speech (AV)	Loudspeakers at every 5°, video projection	PF on coherent location, PSE	10° (T) 19° (U)
André et al., 2014	U	Synchr. Speech (AV)	WFS, 3D projection	PF on coherent location, PSE	18°
Bishop and Miller, 2011	U	Synchr. Speech (AV); McGurk signals (AV); Speech with still face (AV)	Individualized HRTFs for loudspeakers at every 6°, TV	PF on coherent location, PSE	~19°~16°~10°
Lewald and Guski, 2003	U	1 kHz pure tones (A), white diode (V)	Loudspeakers, diodes	9-point scale on common cause 9-point scale on spatial coincidence	~15°~10°
Godfroy et al., 2003	U	Burst of pink noise (A), white flashing circle (V)	Loudspeakers, 2D projection	PF on fusion of sound and vision	~6°

*The “Tr” column details listener training (T, trained; U, untrained; X, unknown). The column “Type of test” lists the applied methods (PF, psychometric function). The “Results” column shows the maximum angle of accepted audio-visual offset*.

Out of the different available indirect measurement techniques, such as emotional judgments or electroencephalography (EEG) measurements and functional magnetic resonance imaging (fMRI) scans, RT measurements offer the possibility to evaluate the perception of audio-visual coherence using realistic speech signals. They have also been used in a variety of neuroscientific tests on cross-modal integration processes and tests using RTs have uncovered pre-attentive and short-lived interference in speech perception (Pisoni and Tash, 1974).

**Figure 1:**
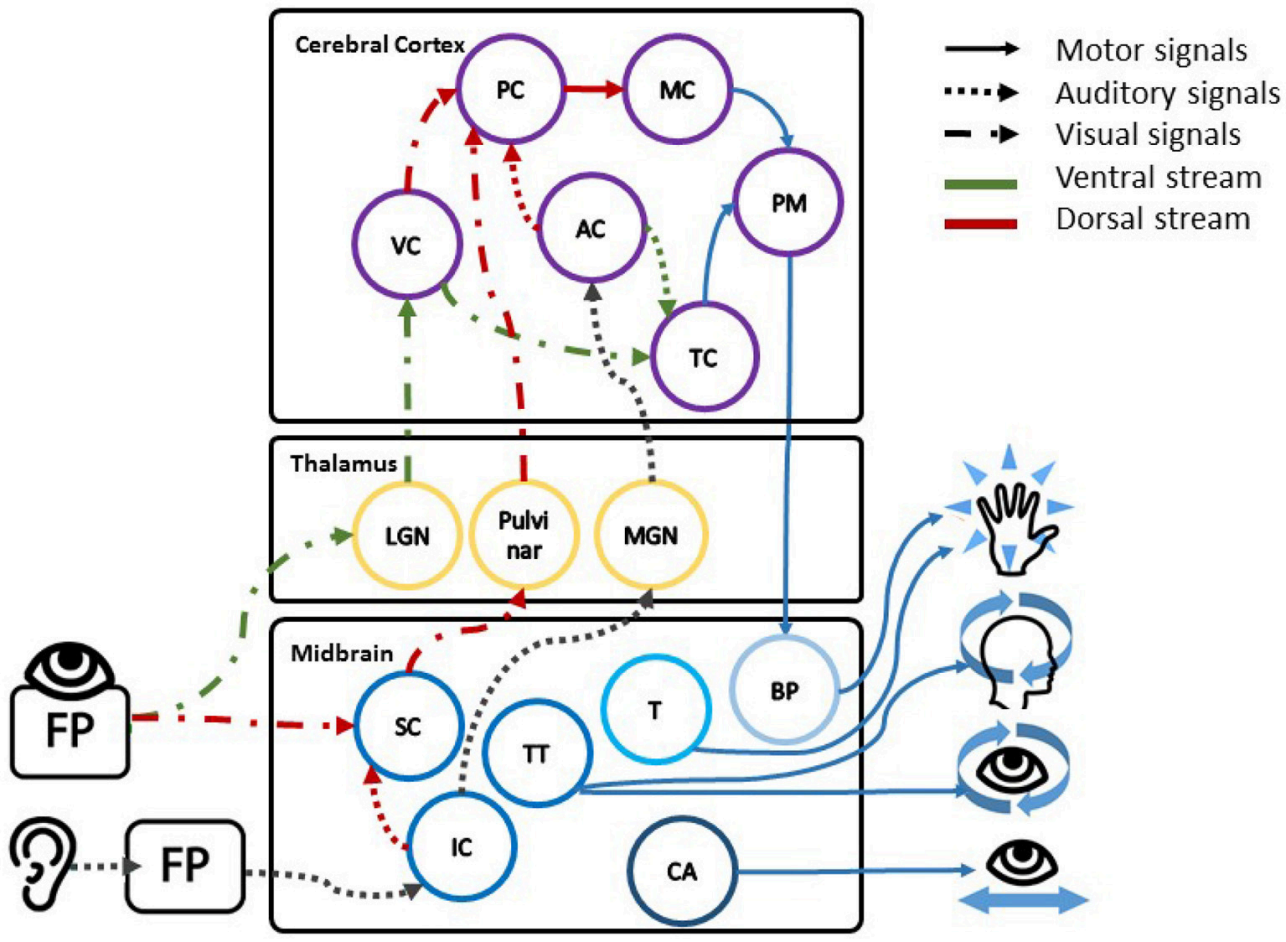
The figure shows the schematic description of the close link between areas in the midbrain processing spatial information, directing movement and the two processing streams. The first stage of combined spatial processing is found in the tectum with the visual spatial information in the superior colliculus (SC); the auditory spatial information in the inferior colliculus (IC); and the direction of head and eye movement in the tectospinal tract (TT). In direct neighborhood the cerebral aqueduct (CA) controls the eye, the eye focus and eyelid movements. The tegmentum (T) on the other side is responsible for reflexive movement, alertness, and muscle tone in the limbs (Waldman, 2009). Following the processing in the midbrain, visual and auditory spatial information is forwarded to the lateral geniculate nucleus (LGN), the pulvinar, and the medial geniculate nucleus (MGN) respectively, followed by the according visual and auditory cortii (VC, AC). Within the two cortii spatial and feature information is separated into the ventral stream (green) across the temporal cortex (TC) and dorsal stream (red) across the parietal cortex (PC). Decisions on motor reaction are then executed by the motor cortex (MC), the premotor cortex (PM), and the basis pendunculi (BP) in the midbrain (Stein et al., 2004; Malmierca and Hackett, 2010).

In order to adopt RT measurements for the assessment of spatial features, the separation of information for different use cases within the brain can be exploited. Research has shown that both visual and auditory information is processed in two main streams within the brain, each fulfilling different functions (Arnott and Alain, 2011; de Haan et al., 2018; Haak and Beckmann, 2018). The ventral stream, shown in green in Figure 1, is known to work on object recognition and analysis of the meaning of the outside world with a close link to memory and consciousness. It is also called the "WHAT"-stream. The dorsal stream or "WHERE"-stream, shown in red in Figure 1, is linked to action responses that are usually conducted subconsciously. These incorporate a wide range of motor responses, encompassing head and eye movements, reaching movements, and also control of the voice. The dorsal stream also includes the superior colliculus in the midbrain as first integration part of auditory and visual spatial information. It is also linked to reflexive head and eye movement, and directing attention to external signals (Stein et al., 2004; Malmierca and Hackett, 2010). These subconscious mechanisms can be used to assess the influence of spatial misalignment on human perception. Tasks can then be designed along one path meanwhile an indirect measure is used to monitor the other path; such as a speech recognition task on the ventral path and RT for the dorsal path under varying spatial offsets. Due to the dual path organization across the brain, no effects are expected along the ventral path as previously shown by Suied et al. (2009): spatial offset did not influence the error rate in an object recognition task. Across the dorsal path, however, subconscious priming of responses, known as the *Simon effect*, and the alteration of *spatial attention* may lead to changes in the RT. These two effects could contribute to describing subconscious processes during the presentation of audio-visual signals with and without spatial offset. Both effects are based on the subconscious interplay of multimodal spatial attention and preparatory movements toward targets (Eimer et al., 2005; Gherri and Forster, 2012).

The Simon effect describes the observation that responses in two-alternative forced-choice-tests (2AFC), in which space is a task-irrelevant parameter, are faster if the stimulus presentation and response side match (i.e., are congruent); responses are slower if the stimulus is presented in the visual hemisphere opposite of the response side, an incongruent response (Lukas et al., 2010; Proctor and Vu, 2010; Xiong and Proctor, 2016). This effect has been measured for visual and auditory tasks, and for responses given with the corresponding fingers from the left and right hands as well as for responses given with the index and middle finger of the same hand (Proctor et al., 2011). The strength of the Simon effect is usually given as the difference in RTs between the congruent and the incongruent stimulus presentations (Proctor and Vu, 2010). The Simon effect has been measured for bimodal signals in the context of divided or unimodal attention, in which responses were only given to the relevant modality, intending a suppression of the irrelevant modality (Lukas et al., 2010; Tomko and Proctor, 2016). Both studies found a cross-modal effect where the Simon effect was elicited by the unattended stimulus. The cross-modal influence of the auditory signal onto responses to the visual stimulus was weaker than the influence of the visual signal on the auditory signal. For realistic stimuli and bimodal perception a Simon effect size of 14 ms has been reported (Suied et al., 2009). Across experiments on the Simon effect, however, the strength of the effect has only been studied with stimuli presented at large symmetric offsets (±30° or headphone presentation). It, therefore, cannot be concluded at which spatial offset the Simon effect starts to be elicited nor whether it changes with increasing offset angles.

In contrast to the Simon effect, the misdirection of *spatial attention* may affect the speech processing. It has been established that auditory spatial perception can direct eye movement and visual spatial attention, especially for sound sources outside the direct field of view (Arnott and Alain, 2011; Alain et al., 2013). In the case of mismatching spatial position of an audio-visual object, such an involuntary eye movement may draw attention away from the attended visual object and thereby alter the bimodal integration process. Especially, speech processing is optimized for bimodal perception (Ross et al., 2007; Ma et al., 2009). This natural integration may be interrupted when the visual signal is not fully perceived. Consequently, the bimodal integration process will be adapted, shifting the weight in the speech processing toward the auditory signal, thus being closer to the unimodal auditory RT.

**Figure 2:**
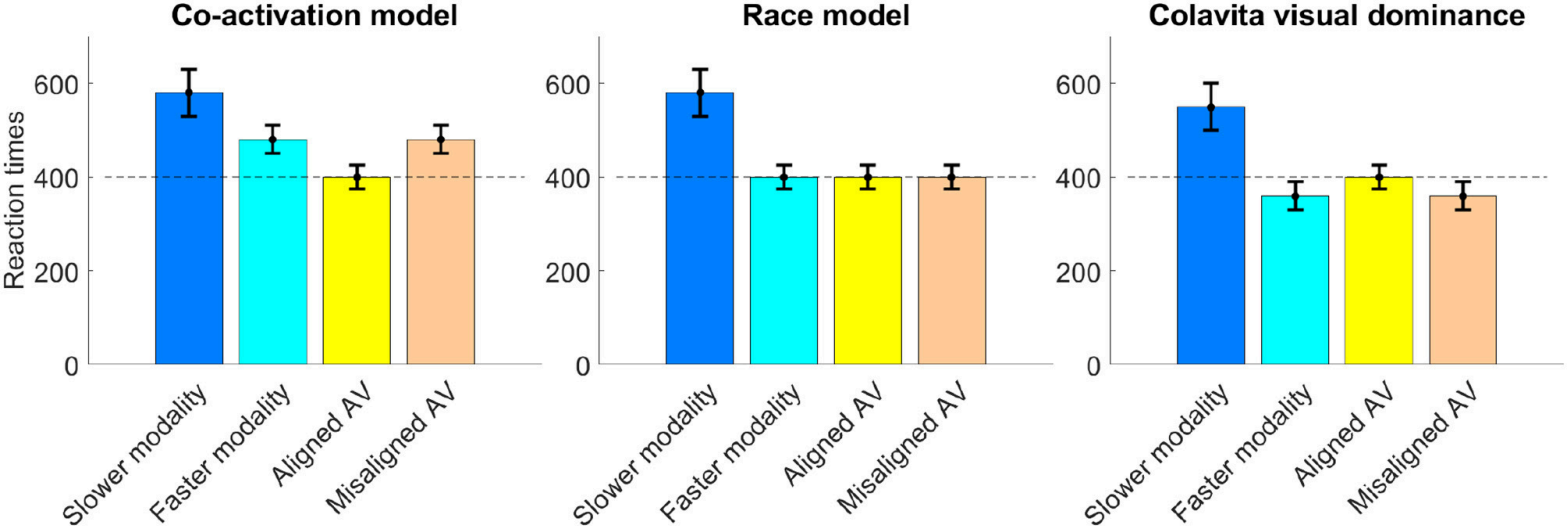
The time course of each of the discussed RT models is depicted showing the relation between unimodal and bimodal RTs, where typically in speech recognition the faster RT is measured in the A condition and the slower RT in the V condition.

In the literature the relationship of unimodal and bimodal RTs following audio-only (A), video-only (V), and audio-visual (AV) stimulus presentation is described by three contradicting models, suggesting that bimodal RTs can be faster, slower, or the same as the faster unimodal one—usually the auditory-only RT in word recognition settings. The expected direction of RT change following the attention shift toward the auditory signal, therefore, remains unclear as shown in Figure 2. In a syllable identification task, for example, RTs were faster for bimodal stimuli compared to unimodal signals (Besle et al., 2004), validating the co-activation model by Miller (1986). This model assumes that there is a statistically significant effect of facilitation in the bimodal condition so that bimodal RTs are faster than either unimodal RT (Miller, 1986). By contrast, an effect of inhibition on RTs with bimodal speech signals is described by Heald and Nusbaum (2014). In a word identification task with either one or three talkers, participants showed slower response times in the audio-visual presentation compared to the audio-only presentation, especially in the case of multiple talkers. This phenomenon is also known as the Colavita visual dominance effect (Colavita, 1974) and summarizes that RTs to audio stimuli slow down in the presence of a visual stimulus, even if participants are specifically required to or would be able to respond to the audio signal alone (Koppen and Spence, 2007). Savariaux et al. (2017), lastly, showed that the detection point of specific syllables varied between different consonants in A, V, and AV conditions, and either followed the stronger modality or a combination of both modalities. For /f/ and /ʃ/, where vision dominates, the bimodal recognition point was significantly slower than the audio-only one, as described by the Colavita effect. For other consonants, however, RT remained as fast as in the faster modality. This effect of equal RTs across unimodal and bimodal conditions is described in the race model (Miller, 1982) and assumes that bimodal signals are processed in parallel. The faster processing chain wins the race and terminates the decision so that the bimodal RT is as fast as the faster modality.

A summary of the multitude of effects is given by Altieri (2010) who compared several different models of RT change. He showed that none of these models can exclusively describe the range of effects in speech recognition when stimuli are degraded (e.g., by noise, or incoherence as with the McGurk-MacDonald effect). Throughout his experiments he also showed that large inter-participant differences existed in the time course and direction of change in RT under varying conditions, leading to contrary distributions of RTs between individuals. These inter-participant differences should thus be accounted for during analysis.

A drawback of RT measurements and the two described effects is that both, the Simon effect and effects of spatial attention, have been shown to decrease in conditions of high perceptual demand. Ho et al. (2009), for example, showed that auditory cuing effects in a visual detection task were suppressed when participants had to concentrate on a rapid visual detection task at the same time. The Simon effect decreased in a study by Clouter et al. (2015) in conditions of high working memory load induced by a 2-back task as compared to a 0-back task. Even though these task-related differences in perceptual demand are not examined in the present study, the perceptual demand may vary in multimedia contexts due to variations in the presented sound scene due to e.g., varying numbers of foreground objects, movement or different acoustic settings. These changes in the background sound scene are also linked to reduced performance in working memory, learning or recall tasks. Haapakangas et al. (2014) showed that a variety of tasks linked to working memory and speech processing were performed worse when interfering speech was presented instead of steady noise, and Ljung (2010), Bockstael et al. (2017), and Nirme et al. (2019) verified worse performance and greater individual effort in speech-related tasks such as learning in multi-talker noise and adverse acoustic conditions. In order to verify the audio-visual offsets obtained through the measurement of RTs for general application in multimedia devices, different experimental conditions will be evaluated.

The present work contributes to research on the understanding of bimodal spatial perception by adopting the indirect measure of RT measurement to investigate the exact offset angle at which an audio-visual spatial offset begins to affect reactions. Even though it has previously been shown that RT measurements differ between spatially matching and mismatching audio-visual stimulus presentation, these methods have not been applied to assess the limits of the ventriloquism effect. RT measurements were chosen to overcome the biases outlined for direct measurements. As no knowledge is gathered about the actual participants' perception through the use of RT measurements, the current experiments will serve to show whether a spatial offset leads to measurable changes in RTs or not. It cannot, however, indicate whether the ventriloquism effect still persists. Following the influence of the background signals on speech processing and RT effects, two experiments are designed to evaluate the test method in two different experimental environments.

The paper is structured as follows. In section 2 the two conducted experiments will be described. The analysis of the RT data in section 3.2 as well as the discussion in section 4 address the Simon effect and the spatial attention effects separately. The findings are collated in the final summary.

## 2. Experiment Methodology

Various mechanisms were discussed to influence RTs following audio-visual stimuli presented with a spatial offset. Two experiments were designed to test whether the outlined effects can be used to study the effect of audio-visual spatial offsets on RTs under realistic conditions. Both experiments used a word recognition task in a 2AFC paradigm, requiring participants to recognize which of two visually indicated words was presented in the audio-visual test signal. The visual signal was presented centrally whereas audio stimuli were presented either centrally or at different offset positions. Audio stimuli were presented directly through loudspeakers to enable natural spatial hearing, and to avoid artifacts and unnatural alteration of localization cues. In the first experiment, pink noise was presented as interfering background signal, whereas a multi-talker speech signal was used in the second experiment. Both sets of results are analyzed in section 3 thereafter.

### 2.1. Experiment One—Pink Noise Interference

The first experiment was conducted to test the effect of audio-visual spatial offset in a condition with pink noise interference. A description of this experiment in combination with tests on unimodal RTs was previously published by Stenzel et al. (2017b). The analysis in this prior publication does not distinguish between the Simon effect and attentional effects but only considers the latter. Furthermore, data are analyzed by modeling the normalized RT distribution with the ex-Gaussian function, the product of an exponential decay with a Gaussian probability density function. Results show different behavior for different groups of participants as defined by the results of the unimodal tests (some bimodally faster, others slower), with the peak of the RT distribution varying significantly between 0° and 5° for participants who were fastest in the audio-only condition.

#### 2.1.1. Experimental Outline and Hypotheses

Given the outlined evidence from the literature on how RTs may be sensitive to spatially misaligned audio-visual signals, the following hypotheses are investigated in the first experiment.

1. An audio-visual offset influences RT in a speech task due to a change in the spatial attention during the onset of the audio stimulus leading to a disruption in the bimodal speech integration process. As a consequence, RTs should tend toward the unimodal RT of the faster modality. As it is not obvious whether bimodal RTs are faster, slower or the same as unimodal RTs as discussed in section 1, it cannot be predicted how RTs will change.
  - Race model. If the race model applies, spatially coherent and incoherent stimuli should not vary, as we would always see the faster RT.
  - Co-activation model. If the co-activation model is applicable, we should see an increase in RTs once the bimodal integration falls apart and the facilitation effect breaks.
  - Colavita effect. If thirdly, the Colavita effect emerges and bimodal RTs are slower than the faster unimodal RT (i.e., showing bimodal inhibition), a break in bimodal integration should lead to speeding up and shorter RTs.

The dominating effect may vary between different participants.

Following this assumption, the same effect should be measurable for offsets to the left and to the right. It should lead to even or axis symmetric changes in RTs along the 0°-line, meaning that data behaves symmetrically across left and right offsets, allowing for pooling across left and right sides. According to the literature on ventriloquism (Komiyama, 1989; Stenzel et al., 2017a), spatial separation for speech stimuli is reliably detected (50%) by trained listeners from 9° offset onwards. No effect at smaller offsets is therefore expected.

2. Changes in RT are induced by an audio-visual offset and can be measured through the Simon effect. It is not evident from literature at which offset angle this effect starts and if it increases with offset. The Simon effect leads to differences between congruent and incongruent responses. The responses must, therefore, be analyzed according to the spatial congruence and incongruence of response key with stimulus presentation, and should then lead to odd changes in RT.

**Figure 3:**
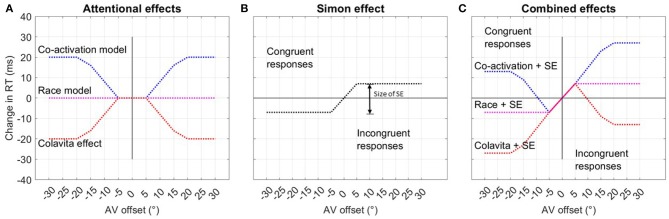
Hypothesis assuming the existence of two separate effects influencing RTs. **(A)** The first graph shows the assumed even, or axis symmetric effect of spatial attention on RTs, differentiating between the co-activation, race, and Colavita models. **(B)** The second graph depicts the odd, or point-symmetric course of RTs due to the Simon effect (SE) for spatially congruent and incongruent responses. The size of the Simon effect is given as the difference in response time between congruent and incongruent responses. **(C)** The last graph summarizes both effects and the expected RT over offset when congruent are summarized under negative offset values and incongruent responses under positive offset values.

The effect of both assumptions is summarized in Figures 3A and B. Figure 3C shows the modeled overall RT distribution assuming purely additive behavior of both effects as indicated in previous research on the interference of the Simon effect and other parameters (Hasbroucq et al., 1989; Hasbroucq and Guiard, 1992; Adam, 2000).

#### 2.1.2. Task

The experiment was designed as an AFC recognition task. It required participants to recognize the keyword presented in the audio-visual test signal (see section 2.1.3) as fast as possible out of two possible choices. Participants were given a control surface with left and right response buttons. On each trial, two words were displayed simultaneously on the screen, one on the left side and one on the right side. The presentation side indicated the corresponding response button: responses for the words on the left side should be given with a left button press, and words on the right side with a right button press. Upon presentation of the audio-visual signal, they were asked to press the button corresponding to the perceived word as quickly as possible. The video was presented centrally and audio was played from one of thirteen loudspeakers covering the range of ±31° in steps of 5°. Word pair and audio offset position were changed pseudo-randomly with every trial; consecutive presentations of the same word pair were not allowed.

#### 2.1.3. Stimuli

The experiment was designed to assess the impact of an audio-visual spatial offset under realistic conditions due to the application of results in media devices. Several audio-only speech corpora exist that are designed to test speech intelligibility under various noise and speech-on-speech interference conditions. Matrix tests were designed to specifically allow for the repeated testing of the same participants avoiding learning effects (Kirsten et al., 1999). A second commonly used test is the Rhyme Test (Fairbanks, 1958) and its derivatives, the Diagnostic Rhyme Test (Greenspan et al., 1998; Voiers, 2005), and the Modified Rhyme Test (House et al., 1963; Brandewie and Zahorik, 2011). In the rhyme tests combinations of monosyllabic words are defined, only differing in the first consonant. The Diagnostic Rhyme Test is specifically designed as 2AFC with consonants of word pairs only differing in one phonetic category. Due to the suitability of 2AFC for RT measurement this test was used as a model for the current RT test design. In addition to the design features of the Rhyme Test, additional care was taken to promote visual speech processing in order to ensure visual attention and strong bimodal integration.

The stimuli were then designed to meet the following conditions.

1. They should be realistic leading to full speech processing in the brain.
2. RTs should be similar between keywords to enable pooling.
3. Audio-visual integration should be ensured.

To achieve this, participants were presented with a realistic speech signal—a keyword embedded in a full sentence—to invoke proper speech processing (McArdle and Wilson, 2008). According to McArdle and Wilson (2008), RTs in word recognition tasks with monosyllabic words mainly depend on the initial and final phonemes. Familiarity only has a minor influence on the recognition time and was therefore not considered in the choice of word pairs. In an audio-only test, Reed (1975) looked at same-different RTs for combinations of consonants with the vowel /a/. Comparing the target-same RTs with the target-different RTs, she found that RTs are longest when only one pronunciation element—manner, voicing, or place—differed between target and distractor. The six most difficult pairs were: (1) /ð/_A_ – /v/_A_; (2) /ð/_A_ – /d/_A_; (3) /ð/_A_ – /θ/_A_; (4) /ʒ/_A_ – /z/_A_ (5) /t/_A_ – /s/_A_; and (6) /ɡ/_A_ – /k/_A_ where we use the subscript A to denote audio presentation. In visual speech recognition, “visemes” are groups of consonants that are formed with the same mouth shape. Inspired by Lidestam and Beskow (2006), five viseme groups were defined. They correspond mostly to the manner categories defined in the chart of the international phonetic association (IPA) and are defined as follows: (1) *bilabial* position for /bmp/_V_ where the mouth is closed and lips are curved in; (2) *labiodental* position for /fv/_V_, showing a closed mouth with the teeth biting the top lip; (3) *interdental* position for /ðθ/_V_ with the mouth in a neutral position and the tongue showing; (4) *palatal* position for /dkɡtsʃ/_V_ showing a neutral mouth; and (5) *approximant* position for /ɾl/_V_ showing a small rounded mouth. Consonants within each viseme group are again harder to distinguish than those from different groups.

In this test, 32 word pairs were chosen, of which each word followed the pattern [consonant]–[vowel]–[consonant] (e.g., “fin”). Each pair only differed in the first consonant and was embedded in the carrier phrase “Say [*keyword*] again.” Pairs were chosen so that the consonants would be from different visemes and would not fall into the group of most difficult audio-only consonant pairs. Videos of the keyword phrases were recorded in a green screen studio with a shotgun microphone pointed at the actor. Two female student actors with British English received pronunciation participated in the recordings. All videos were 2.0 s long, and the keyword was presented at 1.0 s. The videos were recorded in HD 1920 x 1080p, with the codec DNxHD and an MXF wrapper. The audio was recorded at 48 kHz, 24 bit. The edited videos were loudness normalized to −23 LUFS and converted to the ProRes 422 codec. The playback level was set to 60 dB SPL. A pre-test was conducted to find the ten word pairs with the most balanced error rates. In this pretest, three participants performed a 2AFC test in which they were asked to detect the presented word in the audio-visual signal out of two given options. Each word pair was repeated twenty times. The audio and visual stimuli were both presented centrally. The ten word pairs with the highest and most similar scores were then chosen for the final test and are displayed in Table 2.

**Table 2:** Word pairs used in the perceptual test.

**Keywords**		**IPA**		**Phonetic category**		**Viseme category**	
Pong	Song	/p/	/s/	Plosive (U)	Fricative (U)	Bilabial	Palatal
Pen	Den	/p/	/d/	Plosive (U)	Plosive (V)	Bilabial	Palatal
Sin	Fin	/s/	/f/	Fricative (U)	Fricative (U)	Palatal	Labiodental
Can	Fan	/k/	/f/	Plosive (U)	Fricative (U)	Palatal	Labiodental
Cog	Log	/k/	/l/	Plosive (U)	Liquid (V)	Palatal	Approximant
Food	Rude	/f/	/ r /	Fricative (U)	Liquid (V)	Labiodental	Approximant
Beef	Reef	/b/	/ r /	Plosive (V)	Liquid (V)	Bilabial	Approximant
Bus	Fuss	/b/	/f/	Plosive (V)	Fricative (U)	Bilabial	Labiodental
Gong	Wrong	/ɡ/	/ r /	Plosive (V)	Liquid (V)	Palatal	Approximant
Man	Than	/m/	/ð/	Nasal (V)	Fricative (V)	Bilabial	Interdental

*Each word pair consists of two monosyllabic words differing in the first consonant only. The words are grouped according to the viseme and phonetic group of the first consonant (U, unvoiced; V, voiced)*.

#### 2.1.4. Setup

**Figure 4:**
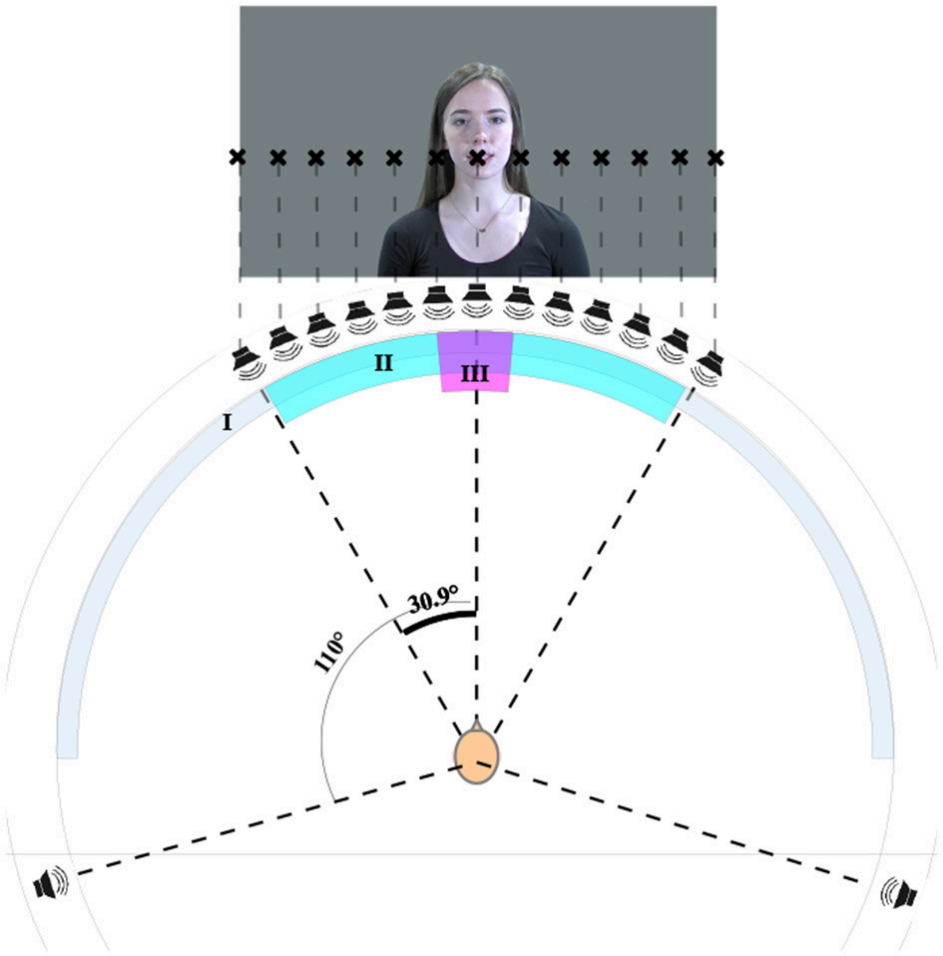
Test setup showing the screen (I), the area covered by the loudspeakers and the video projection (II), and the area covered by the face (III). Written informed consent for publication of the personal image was obtained (CC BY-NC 4.0).

The tests were conducted in an acoustically treated sound booth with an equal reverberation time of 200 ms between 300 Hz and 8 kHz, complying with ITU-T.P.800 in this frequency range (ITU-T, 1996). The thirteen level-aligned loudspeakers (Genelec 8020B) were mounted at approximately ear height on the equator of a spherical structure with a radius of 1.68 m at angular offsets of 0.0°, ±5.1°, ±10.3°, ±15.4°, ±20.6°, ±25.7°, and ±30.9°. These offset angles were chosen as they were the smallest angles possible with the given the size of the loudspeakers and the dimensions of the sphere. For ease of reading, the rounded values will be referred to in the rest of the paper. The image was projected onto a curved, white, acoustically-transparent screen. The video display was aligned to the loudspeakers and the curvature was corrected with the software *Immersive Display PRO* by Fly Elise. The picture covered an area from ±30° left to right at an aspect ratio of 16:9 HD with the center of the picture slightly above the line of loudspeakers. In this way, the mouths of the actors on screen were aligned with the central loudspeaker. The setup is shown in Figure 4. Participants were seated on a fixed chair equidistant from each loudspeaker. The time synchronization of audio and video was enforced by manually delaying the audio signals using a dedicated lip synchronization test signal. The test user interface and the level and delay alignment were implemented in *Cycling '74 MaxMSP 6*. An RME MADIFace XT and RME M-32 DA/M-16 DA were used as the audio interface and for digital-to-analog conversion respectively. The test setup is comparable to the study by Komiyama (1989) as direct loudspeaker feeds at similar positions were used in both studies.

#### 2.1.5. Statistical Design

The variance in RT experiments is usually large compared to the tested difference in means. A sufficient amount of test participants and test items needs to be defined to achieve a statistical power that allows for reproduction of the results (Brysbaert and Stevens, 2018). The current results will be analyzed using a generalized linear mixed effects model (GLME). The simr package in *R* allows for a prior estimation of the statistical power for this type of analysis (Green and Macleod, 2016). Following the tutorial by Green and Macleod (2016), an estimation on the number of participants was run for the hypothesized change in RT across offsets of the co-activation model using the GLME parameters as estimated for the data of three participants (see section 3.2 for further definition of the GLME parameters). In order to achieve sufficient statistical power of 80% for changes in RT of 20 ms, the *powerCurve* function predicted that ten participants would be sufficient. To allow for smaller variations, twenty participants were recruited for the experiment.

#### 2.1.6. Procedure

Each participant performed a learning session prior to the actual RT test. The learning session was designed for participants to become acquainted with the interface, with the task, and with the keywords. It comprised 60 trials, with each of the ten keyword pairs presented six times from a randomly chosen loudspeaker. The main test consisted of 520 trials per participant (10 word pairs × 13 loudspeaker positions × 2 response keys × 2 repetitions) resulting in fourty data points per offset and participant.

**Figure 5:**
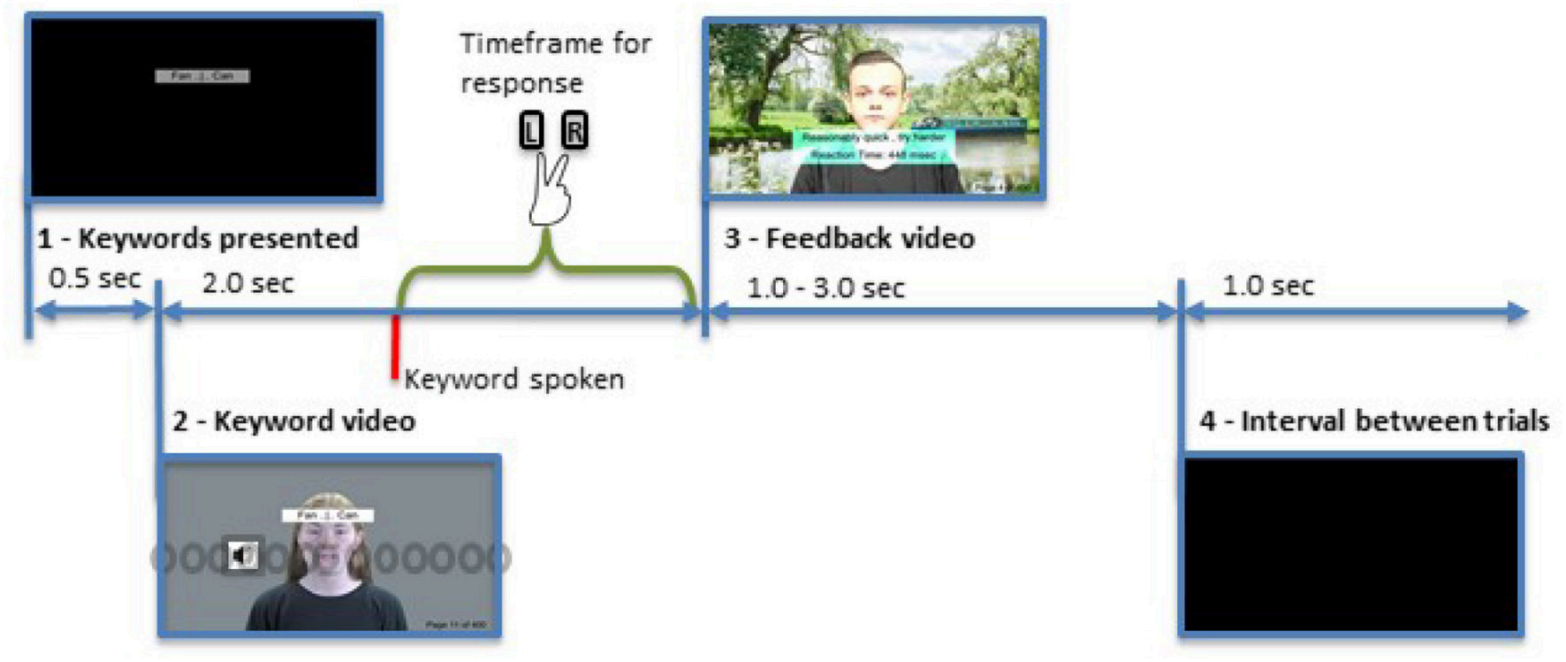
Representation of the trial sequence. Illustrated is a standard trial with audio presented at 10° left. The keywords are presented for 0.5 s, followed by a video of the sentence. The keyword is spoken at 1.0 s within the video. The feedback is presented once a response is given. After an interval of 1.0 s the next trial starts. The gray circles indicate the position of the loudspeakers and the symbol denotes the currently active one. Written informed consent for publication of all personal images was obtained (CC BY-NC 4.0).

The procedure for the test was as follows (and is also visualized in Figure 5).

1. The upcoming two keywords were displayed for 0.5 s.
2. The audio-visual test signal was played (the keywords stayed visible during this presentation).
  - The keyword occurred 1 second into the video.
3. The participant responded.
4. A feedback video was displayed.
  - For correct answers, the RT and a motivating phrase were displayed together with a feedback video encouraging participants to maintain their response speed.
  - For incorrect answers, a feedback phrase and video were displayed.
5. The next keyword pair was then displayed after an interval of 1.0 s.

All feedback videos were spatially coherent (i.e., 0° offset) with audio coming from the center loudspeaker. These videos ensured that the same initial situation was created prior to each stimulus presentation with attention recalibrated to the center, and perceptual adaptation following repeated audio presentations to the same side was avoided. Participants were required to take breaks after 200 and 400 trials.

#### 2.1.7. Response Method

Responses were recorded with two neighboring keys on a *Behringer BCF 2000* musical instrument digital interface (MIDI) studio controller. Participants were free to choose whether they wanted to respond with their index and middle finger of their preferred hand or with the index fingers of both hands but had to stay with one method throughout the test. Proctor et al. (2011) showed that the Simon effect had a similar strength and shape for responses given with the same hand and different fingers, or with the index fingers of the two hands.

#### 2.1.8. Background Interference

Decorrelated pink noise at +10 dB signal-to-noise (SNR) ratio was played from five loudspeakers placed at 0°, ±31°, and ±110° as specified by the the ITU-R (2012) throughout the test. This level was determined in a pre-test to provide approximately equal audio and visual error rates. It alsomasked any specific localizable sound sources within the room such as the projector.

#### 2.1.9. Participants

Twenty participants took part in the test (6 female, 14 male; age 19 to 45 years old; 12 native English speakers; 13 musically trained). They were unaware of the purpose of the study. All participants reported normal hearing and normal or corrected-to-normal vision. Written informed consent was obtained from all participants prior to the study, and the study went through the University of Surrey ethical assessment processes in line with the University's Ethics Handbook for Teaching and Research.

### 2.2. Experiment Two—Speech Interference

The second experiment was conducted to verify whether the results from the first experiments are resilient to changes in experimental conditions, specifically to those leading to a perceptual demand. Experiment one was therefore repeated with multi-talker speech signal introduced as background interferer.

#### 2.2.1. Experimental Design

The stimuli and task in experiment two were the same as in experiment one. In contrast to the first experiment, stimuli were only presented in the range of ±20° to reduce the overall length of the experiment. Again, participants took part in the training session and the main session as outlined above. In this experiment, every keyword was randomly presented twice at each of the eight offsets and four times at 0° leading to a total number of 400 trials. The same feedback videos were used and breaks were scheduled at trial 130 and trial 260.

#### 2.2.2. Statistical Design

Again, a statistical power analysis was performed to determine the number of participants for a statistical power of 80%. As smaller changes in RT are expected in this experiment, a change in RT of 12 ms for the largest offset angle in the co-activation groups was assumed. For this assumption, 20 participants would lead to a statistical power just below 80%. Thirty participants were thus recruited.

#### 2.2.3. Background Interference

Instead of pink noise, multi-talker speech was reproduced as background interference in experiment two. It was composed of eight competing speech signals, with two speech signals presented in each of four loudspeakers placed at ±31° and ±110°. The multi-talker signal was composed in such a way that small speech snippets were intelligible throughout. The overall level of the multi-talker speech signal was kept at +10 dB SNR compared to the target speech signal at 60 dB SPL.

#### 2.2.4. Participants

Thirty participants took part in the experiment (14 female, 16 male; age 19 to 65 years old; 16 native English speakers; 18 musically trained). They were unaware of the purpose of the study. All participants reported normal hearing and normal or corrected-to-normal vision. Written informed consent was obtained from all participants prior to the study, and the study went through the University of Surrey ethical assessment processes in line with the University's Ethics Handbook for Teaching and Research.

## 3. Results

In section 2, experiments to investigate the effect of spatially coherent and incoherent audio-visual signals on word recognition times in two different noise environments were described. The analysis will look at the percentage correct as an indicator of task difficulty first. The RTs will be analyzed separately for each identified effect per experiment and in comparison between both tests. The analysis data is undertaken to determine the spatial offset angle at which RTs change significantly compared to coherent presentation, and to define the effect of different background noise, causing higher cognitive load on the overall results.

Prior to any analysis, data from both tests were trimmed by removing extreme RTs below 150 ms and above 1100 ms, corresponding to 0.1% of data points in experiment one and 0.3% in experiment two. In both tests, a number of participants asked about the purpose of the test after having conducted the test. No participant reported becoming aware of the audio-visual offset at which stimuli had been presented.

### 3.1. Percentage Correct

The percentage of correct responses per test, participant, offset, and Simon effect offset was calculated and reached mean values of 92.47% for 10385 responses in test one and 92.24% for 11970 responses in test two. A repeated measures ANOVA showed that, within each test, percentage correct did not vary significantly between different offsets [*F*_*test*1(12)_ = 0.96, *p*_*test*1_ = 0.45; *F*_*test*2(8)_ = 0.74, *p*_*test*2_ = 0.66] or between congruent and incongruent responses [*F*_*test*1(2)_ = 0.40, *p*_*test*1_ = 0.59; *F*_*test*2(2)_ = 0.2, *p*_*test*2_ = 0.82]. As there was no significant difference between the congruent and incongruent responses, no further analysis of the Simon effect per offset angle was carried out. Furthermore, no significant difference existed in the error rate between the two test conditions with *F*_(8)_ = 0.50 and *p* = 0.86. This indicates that the spatial offset did not significantly influence the word recognition in either condition of perceptual load. Neither did the multi-talker speech interferer result in a significant decrease in performance. This may be due to the relatively low signal to noise level of 10 dB SNR, allowing participants to clearly understand the spoken words on both tested conditions.

### 3.2. RTs

In the analysis of the RTs, only correct responses were considered. The contributions of the spatial attention effect and the Simon effect were analyzed separately. For the evaluation of attentional effects, data were pooled across left and right offsets; for the analysis of the Simon effect, congruent or same-side responses for left and right key responses were pooled under negative offset values and incongruent or opposite-side responses were pooled under positive offset angles, by inverting the sign of the offset for right key responses. For the statistical analysis, the generalized linear mixed-effects model (GLME) was used as proposed by Lo and Andrews (2015), followed by tests on the *F*-statistics. This methodology is necessary as RT data is not normally distributed but has a strong positive skew. The distribution of RT data was modeled by a Gamma function as it yielded slightly better Akaike information criterion (*AIC*) and Bayesian information criterion (*BIC*) fits than the inverse Gaussian distribution for each set of test results. The link function within the GLME describes the interplay of underlying effects. According to Lo and Andrews, the identity link best describes the additive behavior of several effects on RTs as a change in one test parameter directly influences the RT. Across all analyses, the GLME was used with the following setting: Trial + Offset + Response Hand were defined as fixed effects. Trial refers to the sequential count of the given answer, and Response Hand refers to the left or right button press. As described in the experimental design, some participants responded with two fingers on the same hand, whereas others used the index finger of left and right hands. The influence of these fixed effects is given as difference in the mean and standard error (SE)—the statistical inference of the true position of the mean based on the standard deviation and the distribution of the data. Keyword + Participant were defined as random effects, again following the suggestion of Lo and Andrews (2015). The change in RT due to these parameters is given as standard deviation (SD), the variance unexplained by the model. Model fits were compared between modeling with and without each of these two random factors. In all cases model fits were higher with random factors included.

### 3.3. Experiment One—Pink Noise Interference

The first experiment examined word recognition rates with pink noise as background interferer. The noise signal was presented uniformly from five loudspeakers at 10 dB SNR.

#### 3.3.1. Attentional Effects

As outlined in section 2.1.1, it was hypothesized that the attentional effect equally affects offsets to the left and right side. This statement was validated by pairwise comparing RTs from offsets toward both sides. The GLME was fitted for each offset angle with Trial + Response Hand + Side as fixed effects and Keyword + Participant as random effect. RTs varied in the range of less than ±1 ms between the two sides. Even though the difference was significant at 5° offset (*p* = 0.03) and 10° offset (*p* = 0.01), no consistent trend across all offsets was observed. When pooling across all offsets on each side, no significant difference was measured. For that reason, data from left and right side was pooled for the following analysis of attentional effects on RTs.

RTs in this experiment averaged around 447 ms (*standard error (SE)* = 11 ms). The analysis of the *F* statistics on the output of the fitted model showed that the parameters Trial and Response Hand reached significance with *p* < 0.01, and *F*_*Trial*_ = 119.4 and *F*_*Response Hand*_ = 133.6 respectively. This shows that learning took place and RT decreased with every trial by 0.06 ms (*SE* = 0.005 ms), summing to give a mean reduction in RTs of 33 ms between first and last trial. Differences between response hands averaged at 19 ms (*SE* = 1.6 ms) with faster right key presses. The Offset did not influence RTs significantly with *p* > 0.1. The random factors Participant and Keyword lead to standard deviations in RTs of 36 and 28 ms, respectively.

**Figure 6:**
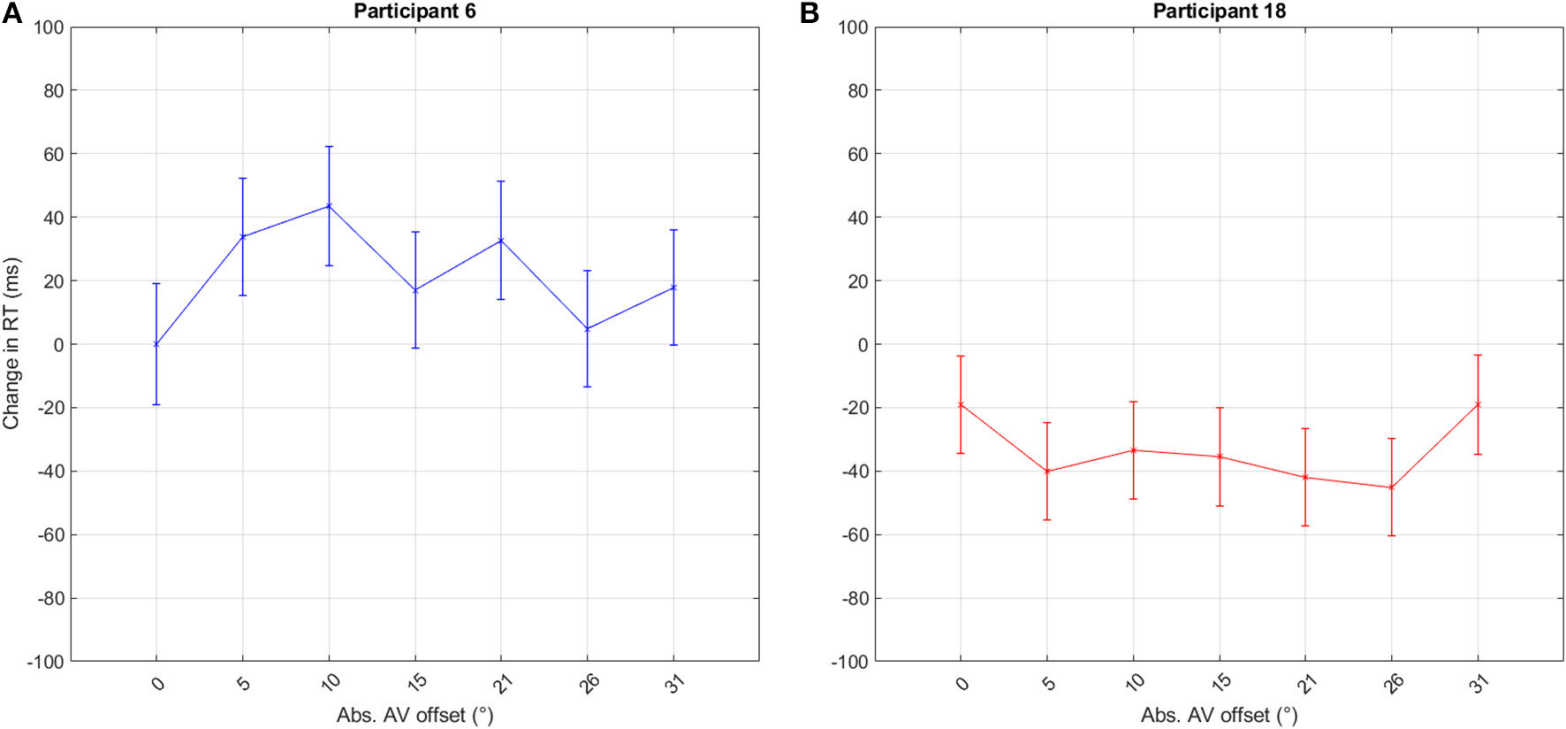
RT distribution of two participants from the first experiment when pooled across left and right offsets. The graphs show the estimated difference in RT from the mean RT at 0°. The bars indicate the standard error. These two datasets exemplify the huge differences between participants and also show both typical co-activation model **(A)** and Colavita effect **(B)** at offsets. The y-axis indicates the difference in RT between responses given with no offset, 0°, and those with offset.

As outlined in section 1, a change in visual attention may impact RTs differently for different participants. Figure 6 visualizes the large variation among single participants showing a typical case of the co-activation model (Figure 6A) and of the decreased Colavita effect at offsets (Figure 6B). These differences may add up in misleading summation effects.

For this reason, the GLME analysis was performed for each participant separately comparing data from 0° to that from all other offsets. In this way, the general trend across RTs at offsets can be summarized per participant. Afterward, participants were grouped according to the resulting *t*-statistics. A *t*-value of 0.675 or *p* = 0.5 was chosen as grouping criterion indicating an above chance for a difference between RTs for coherent vs. incoherent presentation. A value of *t* > 0.675 was assumed to be an indicator for the co-activation model, with values at offsets slower than at 0°; a value between *t* < = 0.675 and *t* > = −0.675 was used as an indicator for the race model; and a value of *t* < −0.675 was linked to the Colavita effect, where responses with good bimodal integration are slower than those from a disrupted, or auditorily dominated perception. Four participants fell into the first group, eleven into the second group, and five into the third group.

**Figure 7:**
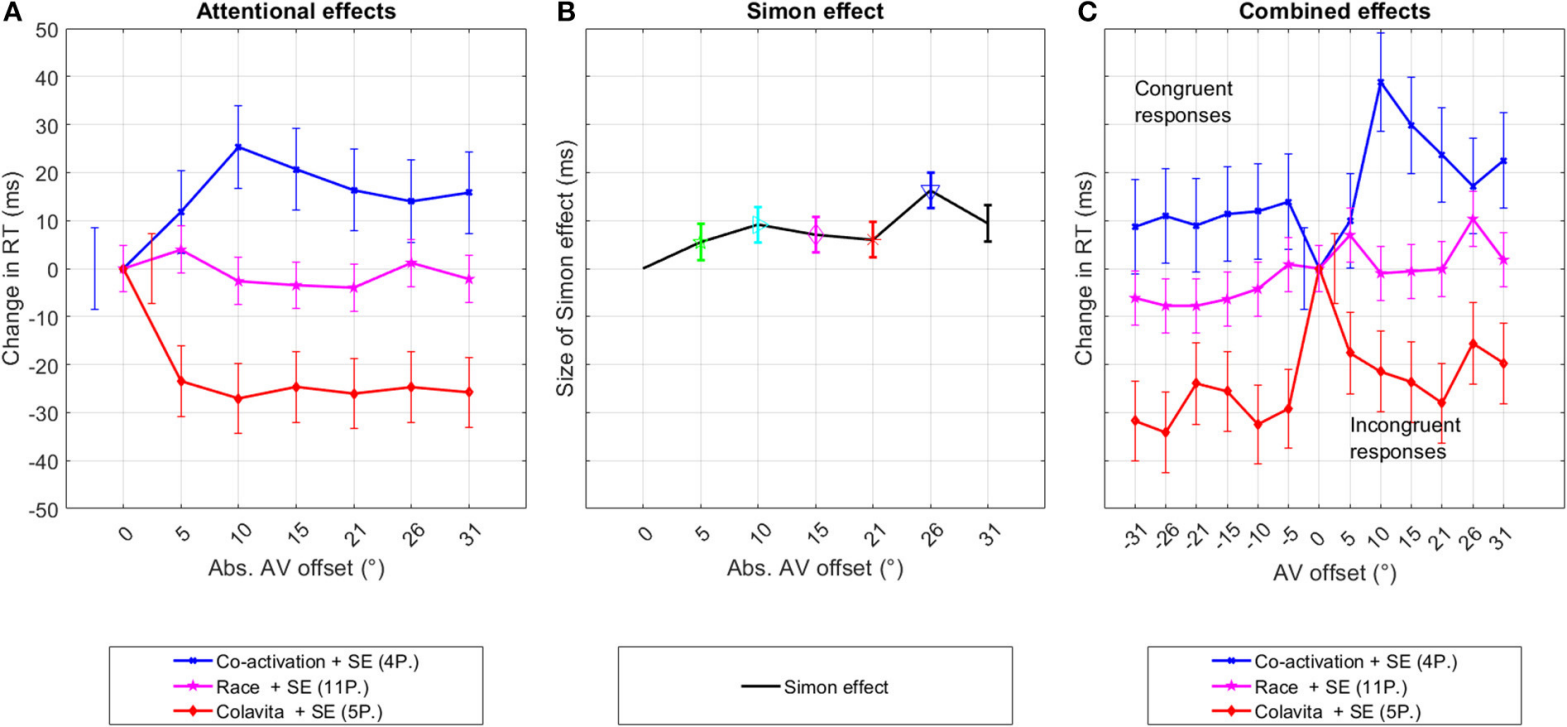
Experiment one—pink noise. This figure shows the decomposition of the original RT distribution into the underlying even and odd components per participant group that were possibly caused by changes in the spatial attention and the Simon effect. The third graph depicts the original distribution of the data sorted into congruent and incongruent responses. The graphs show the changes in mean RTs between data from 0° and data at offsets in **(A, C)**, and as the difference in RTs between congruent and incongruent responses **(B)**. The bars indicate the standard error.

In the co-activation group, Offset did not change RTs significantly [*F*_(6,1897)_ = 1.7, *p* = 0.12], with a pairwise comparison showing that RTs at offsets 10° and 20° were significantly slower than RT at 0° (*p* < 0.5). At all other offsets, RT was also slower but did not reach significance. In the race model group, no significant variation in RT was measurable [*F*_(6,5333)_ = 1.0, *p* = 0.42]. Participants linked to the Colavita effect answered significantly faster at all offsets compared to 0° [*F*_(6,2346)_ = 2.7, *p* = 0.012]. The course of RTs across offsets for the different groups, as well as the confidence intervals, are shown in Figure 7.

A further Fisher exact test was performed to test whether a relationship exists between participants' musical training and the defined RT groups. No significant overlap between the two groupings was observed with *p* = 0.117, and 27 tables evaluated. Furthermore, the GLME was repeated with musical training as additional fixed effect. The results showed no significant difference between participants with and without musical training (*p* > 0.1).

A GLME was fitted to the grouped RT data of participants. The general trend of participants' RTs to decrease, stay constant or increase with offset was defined as grouping criteria. The two participant groups of increasing and decreasing RTs with offset resulted in significant differences between RTs at 0° and other offset angles. These results indicate that approximately 50% of participants respond to a spatial offset introduced by an audio signal with altered RTs. In contrast to the hypothesis, these participants who are sensitive to audio-visual offsets, respond to spatial offsets as small as 5° rather than the predicted 15° to 20° offset angle.

#### 3.3.2. Simon Effect

The Simon effect is given as the difference in RT between congruent and incongruent responses. The GLME was fitted to the data comparing the difference in RT between each pair of offset angles. Data from 0° offset was omitted from the analysis. The difference between congruent and incongruent responses was stable between 5 and 9 ms at all offsets except at 26° offset where it increased to 16 ms. Significant differences between congruent and incongruent responses were reached at 10° [*F*_(1,1487)_ = 4.7, *p* = 0.03], 26° [*F*_(1,1469)_ = 4.7, *p* < 0.01], and 31° [*F*_(1,1470)_ = 4.7, *p* = 0.02].

When data were pooled across adjacent offset angles, the Simon effect significantly affected RTs at all offset positions [5° – 10°: *F*_(1, 2961)_ = 5.7, *p* = 0.02; 15° – 20° : *F*_(1, 2952)_ = 4.8, *p* = 0.03; 26° – 31° : *F*_(1, 2934)_ = 19.4, *p* < 0.01].

Overall, the Simon effect was measurable but weak, leading only to significant results when data from adjacent offset angles were pooled. Similar to the attention effect, significance was reached for the first pooled group for data from 5° to 10° indicating that small offset angles influence manual responses.

### 3.4. Experiment Two—Speech Interference

The second set of experiments was conducted to validate RTs as test method under circumstances of higher perceptual demand. Experiment one was therefore repeated with multi-talker speech as the interferer. Following the previous results, only audio-visual offsets up to 20° were tested.

#### 3.4.1. Attentional Effects

Similar to experiment one, no significant differences between left and right side responses were observed in a general GLME. Data from both sides were therefore pooled per measured offset angle. The analysis, in general, follows the procedures as outlined for experiment one. In the second experiment with speech as the interfering background signal, the GLME showed an average RT of 501 ms (*SE* = 11.6*ms*). The strongest effect on RT was linked to a decrease in RT by 0.11 ms per trial due to learning adding up to a total decrease of 44 ms across a test run [*F*_(1,11032)_ = 195.0, *p* < 0.01]. It was followed by Response Hand [*F*_(1,11032)_ = 9.8, *p* < 0.01, *RT*_*dif*_ = 5.6*ms* (*SE* = 1.8*ms*)]. The random factors Participant and Keyword resulted in standard deviations in RTs of 52 and 27 ms, respectively. The effect of Offset did not reach significance.

**Figure 8:**
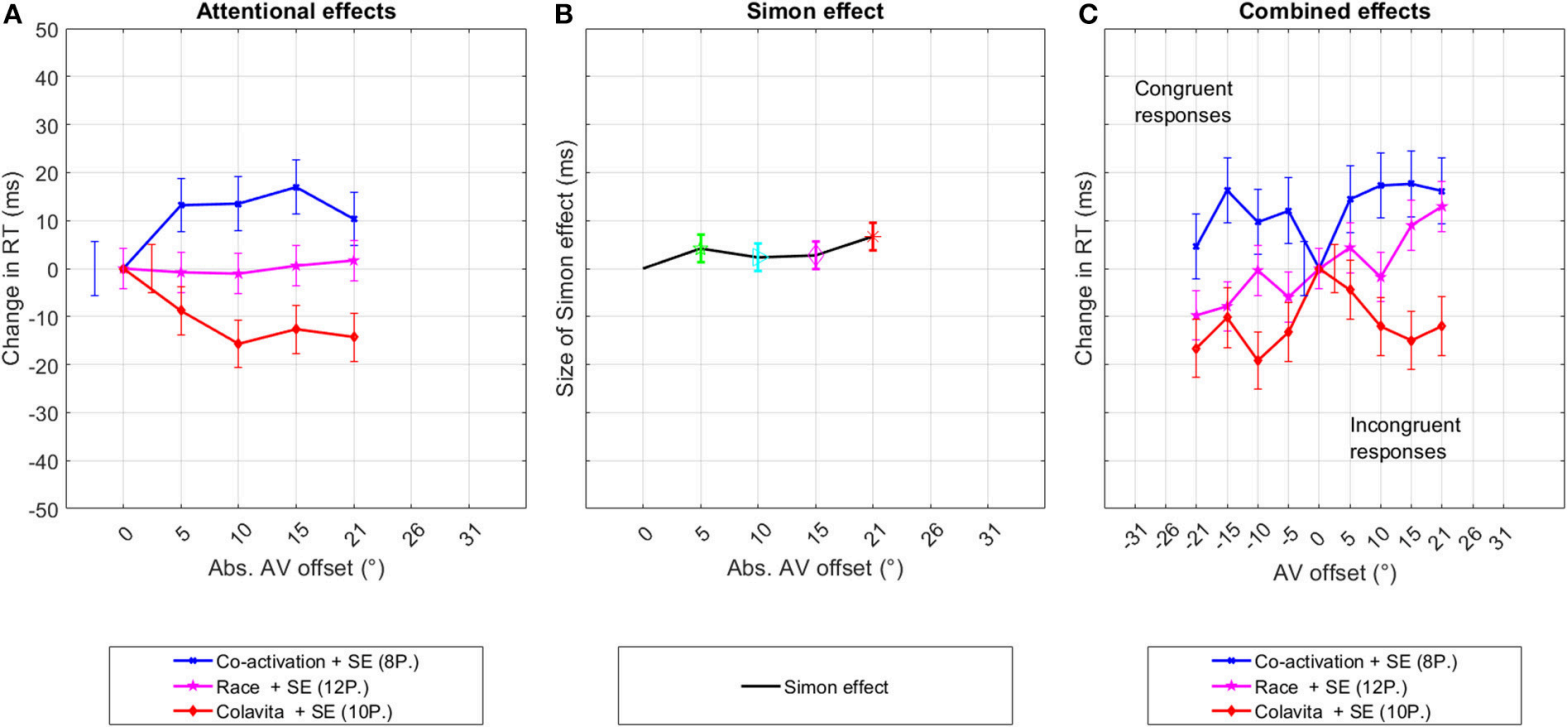
Experiment two—multi-talker speech interference. This figure shows the decomposition of the original RT distribution into the underlying even and odd components per participant group that were possibly caused by changes in the spatial attention and the Simon effect. The third graph depicts the original distribution of the data sorted into congruent and incongruent responses. The graphs show the changes in mean RTs between data from 0° and data at offset in **(A,C)**, and as the difference in RTs between congruent and incongruent responses **(B)**. The bars indicate the standard error.

Following the reasoning in section 3.3.1, participants were again grouped into three groups. Eight participants were assigned to the co-activation group according to their individual *t*-value. Within this group, Offset had a significant effect on RTs [*F*_(4,2956)_ = 2.7, *p* = 0.03] with significantly slower RTs at 5°, 10°, and 15° in the pairwise comparison to 0°. Twelve participants were linked to the race model. RTs from this group did not vary significantly between any offset [*F*_(4,4412)_ = 0.1, *p* = 0.97]. Ten participants had *t*-values below −0.675, indicating a speeding up with offset. In this group, RTs were significantly faster [*F*_(4,3654)_ = 3.1, *p* = 0.01] at all offsets compared to 0°, except at 5° (*p* = 0.08). Results from the grouped analyses are shown in Figure 8 as differences in RTs between data from 0° and the according offset angle.

Again, a Fisher exact test was performed to test whether a relationship exists between participants' musical training and the defined RT groups. No significant overlap between the two groupings was observed with *p* = 0.727, and 78 tables evaluated. The GLME was also fitted with musical training as additional fixed effect showing no significant difference between participants with and without musical training (*p* > 0.1).

The analysis of data from the second experiment, in which a condition of a higher cognitive load was created, confirmed that an audio-visual offset can still affect RTs in this condition. Similar to the results from experiment one, participants could be classified into three groups. Significant changes in RTs between 0° and 5°−10° offset were measured for the first and third group. Similar to experiment one, this offset angle is smaller than hypothesized. In the first experiment 50% of participants belong to one of these groups, whereas in the second experiment, 66% of participants belong to one of these two groups.

#### 3.4.2. Simon Effect

For the analysis of the Simon effect, the same analyses were repeated as described for experiment one in section 3.3.2. Significant differences between RTs of congruent and incongruent responses were reached at 5° [*F*_(1,2187)_ = 4.5, *p* = 0.03, *RT*_*dif*_ = 8*ms*] and 20.5° [*F*_(1,2199)_ = 11.3, *p* < 0.01, *RT*_*dif*_ = 13*ms*]. When data were pooled across adjacent offsets, significance was reached in both cases with *F*_(1, 4417)_ = 5, *p* = 0.02, *RT*_*dif*_ = 6*ms* at 5°–10°, and *F*_(1, 4407)_ = 11.1, *p* < 0.01, *RT*_*dif*_ = 9*ms* at 15°–20°.

#### 3.4.3. Comparison Between Experiment One and Experiment Two

The *F*-statistics of the GLME model fitted on the difference between experiment one and experiment two shows that there is a significant difference [*F*_(1,17687)_ = 8.8, *p* < 0.01] between the two experiments. Responses in experiment two with speech background interference were on average 39 ms (*SE* = 13*ms*) slower than those in experiment one with a static noise interference.

## 4. Discussion

RT data from a word recognition task was collected for stimuli presented at 0° to 31° audio-visual spatial offset. The experiments served to identify the spatial offset at which RTs are significantly affected. Data were analyzed according to the two identified effects: alteration in visual spatial attention and the Simon effect. The analysis showed that for both effects significant differences in RTs were measurable between 0° and 5° to 10° offset. These results will be discussed along the lines of hypothesized behavior for each effect, followed by a discussion on the implications in the wider context of the ventriloquism effect and media applications.

### 4.1. Spatial Attention and Speech Integration

As outlined in section 1, it was assumed that interruption in bimodal integration caused by changes in spatial attention can lead to various changes in RT. Considering the integration of speech, three possible theories were elaborated on the direction of change in RTs. The subsequent analysis showed that 30–50% of participants fell into the group linked with the race model, showing no significant variations in RTs across offsets. The responses of the other participants were described by either the Colavita visual dominance effect or the co-activation model, each leading to significant differences in RTs from 5° onwards.

The race model theory states that bimodal RTs are always as fast as the fastest unimodal RTs. A change in the bimodal integration process would therefore not be reflected in a change in RTs. The RT analysis of spatial attention effects supports this theory. RTs across all participants, and the specific RTs of 50% of participants in experiment one, and 30% of participants in experiment two did not reveal any significant variation of RTs with spatial offset. Apart from the race model theory, these findings are supported by research on auditory speech processing, which has shown, that audio-only speech comprehension does not depend on the specific auditory spatial attention. Alsius and Soto-Faraco (2011), for example, conducted a searching task, in which participants had to either detect or localize whether one of the presented speech stimuli matched the concurrent video; two to four speech stimuli were presented simultaneously. Response times did not vary in the detection task with an increasing number of presented voices but did increase in the localization task. Tests on the McGurk effect have revealed similar results: Bishop and Miller (2011) showed that the strength of the McGurk effect is not affected by an audio-visual offset as long as attention is paid to the visual signal (Andersen et al., 2009).

In summary, these findings suggest that a range of cognitive processes such as speech processing do not depend on the spatial alignment of the co-occurring unimodal signals. This finding is supported by the two streams in the brain where feature and object information, such as speech, is monitored across the areas linked to the ventral stream and spatial information is handled in a different stream.

In contrast to this argument, the majority of participants was affected by the offset in their bimodal integration when considered separately, either slowing down or speeding up significantly in their responses as predicted by the co-activation model and the Colavita visual dominance effect. Both effects were hypothesized to be the result of a degradation on the visual input due to misguided visual attention. Apart from the current example of speech processing, affected by misguided visual attention, further examples can be found in the literature. Saccadic movements (eye movements) toward a visual target, for example, slow down when a spatially disparate audio signal is simultaneously presented (Diederich and Colonius, 2004). Additionally, Spence and Driver (1997) showed that reactions to visual signals are faster when primed by a spatially matching audio signal as opposed to slowing down when the audio signal is presented at a separate spatial location. Arnott and Alain (2011) go even further, stating that “*the major function of auditory localization was to direct the eyes to a location of interest.”* The current findings in combination with further examples from research suggest that, for some participants, the audio target in the current experiments may have attracted attention away from the central visual signal and thereby altered the bimodal integration process. This assumption seems evident given the close link between subconscious bimodal spatial processing in the superior colliculus and its role in head and eye direction (Waldman, 2009). For validation of this hypothesis, however, eye movements will need to be tracked in future experiments.

The analysis of attentional effects showed that variations across different participants exist with no one model favored across the two experiments. Given a fixed setting, it appears as if different people operate differently in the situation. This finding can be seen as further evidence to the results presented by Altieri (2010) who revealed great individual differences in handling degradations in bimodal perception.

In combination, the hypothesis was confirmed that an audio-visual spatial offset can affect participants' perception, but individual differences exist.

### 4.2. Simon Effect

The current experiments revealed a significant Simon effect in a condition of highly merged senses—realistic speech signals presented over loudspeakers with a centrally presented visual signal. With differences between 6 and 13 ms, the measured size of the Simon effect is in the range of results by Suied et al. (2009), who found differences between congruent and incongruent responses of 12 ms due to the Simon effect. As in the current study, realistic stimuli were used, and audio signals were presented at 0° and 40° offset on loudspeakers, allowing for natural localization cues. Lukas et al. (2010) and Tomko and Proctor (2016) measured slightly larger values with 14 and 20 ms, respectively. In these experiments, artificial stimuli such as a 400 Hz tone were used. Audio signals were presented on headphones only and the visual signal was presented to the left and right side. These major differences between the test setups may be responsible for the variation between results.

None of these previous experiments on the Simon effect investigated the relationship between the strength of the effect and the size of the offset. The present results show that the size of the Simon effect is similar for audio-visual offsets between 5° and 20° in experiment one, and 5° to 15° in experiment two. The effect size measured by Suied et al. (2009) at 40° audio-visual offset is within the range of differences in RTs as found in the current experiments. Therefore, it is concluded that the Simon effect is measurable from small offset angles onwards and that the size of the Simon effect does not increase or vary otherwise with increasing audio-visual offset.

### 4.3. Influence of Speech vs. Noise Interference

Experiment one and experiment two were performed to investigate whether different background distractors, in particular, speech interference, would affect the results; it was hypothesized that the interfering speech signal introduced in the second experiment would lead to smaller effects sizes due to a higher perceptual demand. This hypothesis is supported by the overall increase in RTs in experiment two compared to experiment one, with mean RTs being 39 ms slower in the experiment with interfering speech. For both the Simon effect and spatial attention effects, a decrease in the effect size was measured. Furthermore, the learning effect in experiment two was greater than in experiment one as indicated by the parameter Trial. Even though the second experiment was shorter (400 trials) than the first one (520 trials), the statistical effect size was larger [*F*_(1,8828)_ = 144.08 compared to *F*_(1, 8864)_ = 114.76], and differences per trial were approximately 60% higher. Expressed in time, RTs increased by 0.11ms per trial in experiment two compared to 0.06ms per trial in experiment one, adding up to a total difference between first and last run respectively in RTs of 40 ms in experiment two and 31 ms in experiment one. The word recognition as such, however, was not affected by the higher load condition as indicated by the constant percentage correct. The speech background, therefore, required greater adaption such as the suppression of unnecessary information but did not interfere with the speech processing. The impact of competing speech as opposed to competing noise at similar SNRs was already shown in a number of publications. Distracting speech signals at only 10 dB SNR, for example, led to a distraction value of 4 out of 5 points and resulted in a degraded performance in an operation span task and longer RTs in an n-back task (Haapakangas et al., 2014).

An interesting effect of the presentation of competing speech signals is the resulting decrease in localizability. Kopčo et al. (2010) showed that localization errors increased by up to 36% when competing speech signals were presented. The increase in error was greatest when target and masker sounds were presented at an offset of 10° compared to larger offsets. It is hypothesized that the multi-talker interferer in the second experiment, presented at ±31° and at ±110°, resulted in a decreased localizability of the target speech at larger offsets. The perceived overall location of an audio-visual stimulus is defined by the Bayesian integration of the relative localizability of each unimodal stimulus (Alais and Burr, 2004; Godfroy-Cooper et al., 2015). When localizability of the audio stimulus is reduced, the dominance of the visual signal will be stronger. This effect is evident in experiments on saccades (rapid eye movements) (Diederich and Colonius, 2004). The speed with which eyes move toward a visual target depends on the perceived distance between the audio and visual signals. With a smaller perceived distance in noisier conditions, eye movement toward the visual target is less strongly distracted by the interfering stimulus (Diederich and Colonius, 2004). These findings can also be consulted to explain the smaller effect sizes in the multi-talker speech condition.

The lack of impact on speech recognition is not surprising, as speech intelligibility starts to be affected at SNRs smaller than 0 dB (Cooke et al., 2013).

In general, the effectiveness of RT measures to define the offset angle at which an audio-visual offset affects perception has been validated in two experimental conditions. The measured differences in RTs between matched and spatially mismatched presentation were smaller in the condition of the higher cognitive load. However, in both conditions differences in RTs occurred from 5° onwards.

### 4.4. Conclusion and Implication for Audio-Visual Applications of Both Effects

The current studies were conducted to find the limits at which an audio-visual offset can be perceived. These limits are of interest to the multimedia industry, working on immersive technologies, which aim at recreating surrounding sound scenes and 3D images in a realistic and convincing manner.

The results suggest that spatial mismatches as small as 5° are processed in subconscious brain areas across the dorsal stream and lead to response-priming and possible changes in spatial awareness. This offset angle is smaller than PSEs reported for speech signals in direct measurements of the limit of ventriloquism, lying between 9° for participants with musical training and 19° for those without any auditory training (Komiyama, 1989; Stenzel et al., 2017a) (see Table 1). In the direct tests, PSEs were generally larger for ecologically valid signals, in particular speech, compared to other signals, with reported PSEs of 4° to 6° for noise type signals. Studies on temporal ventriloquism, for example, confirm this notion that speech allows for greater variation than other signals (Vatakis and Spence, 2008). It is assumed that these effects on direct measures are the result of a special binding for signals sharing the same or linked temporal features (Spence and Soto-Faraco, 2012). This binding effect does not seem to influence the action processing or the subconscious localization integration. We speculate that the measured offset of 5° here hence is valid not only for speech but for all types of audio-visual signal.

Further support for this assumption comes from the observation that no link between musical training and participants' sensitivity to the spatial misalignment could be established: the current experiments show that similar and more critical results to those of trained participants from direct measurements can be obtained with a mixture of trained and untrained listeners when RTs are used as an indirect measurement tool. This difference in results between direct and indirect measurement methods indicates that both trained and untrained participants are affected by audio-visual offsets in a similar way, but it suggests that only musically trained participants who are experienced in making auditory judgments are able to consciously access this information.

Whether these subconscious processes also lead to a break down of the ventriloquism effect, and to what extent they influence the perceived realism in a virtual environment cannot be concluded from these results. The speech intelligibility as such, for example, was not affected by a spatial mismatch between audio and visual signals as error rates did not increase. Yet, experiments with film excerpts have already shown that spatially aligned audio-visual presentations are preferred compared to spatially static presentations as shown by Maier (2009) and Hendrickx et al. (2015). In both studies, excerpts of feature films and footage of a performing orchestra were presented either with a spatially coherent or incoherent audio mix. Participants had to judge which audio mix was more suitable for the presented video. In both studies, coherent mixes received greater preference ratings.

In the majority of literature on bimodal spatial effects, it has been shown that the effect of changes in the visual signal on the overall results is by far greater than spatial changes in the audio signals. For this reason, an experimental setup in which the spatial offset is induced by the visual signal with static audio signals should be investigated. Such a scenario is also motivated by the current practice of presenting speech signals in the center loudspeaker in cinematic productions.

### 4.5. Summary

The current experiments were conducted to elaborate RT measurements for the definition of the audio-visual spatial offset affecting perception under two conditions of cognitive load. This method of measurement was chosen to overcome the variety of factors influencing direct measurements, leading to a large spread in measured perceivable offset angles. The results validated the hypothesis that subconscious mechanisms across the dorsal stream can be used to study the effect of spatial offset on perception. In both experiments, participants showed response primings due to the audio-visual offset. Differences in RTs between congruent and incongruent stimulus presentation were measured for an audio-visual offset from 5° onwards. These differences were measured for the two effects under investigation: the Simon effect and effect on spatial attention. The results show that an audio-visual offset of 5° and more interferes subconsciously with action processing, suggesting that audio and visual spatial information along the dorsal stream is not merged at this offset angle. The method of RT measurements, however, only gives significant results for two out of the three groups of participants.

## Ethics Statement

Written informed consent was obtained from all participants prior to the study, and the study went through the University of Surrey ethical assessment processes in line with the University's Ethics Handbook for Teaching and Research.

## Author Contributions

HS: design, facilitation, setup of the experiments, full analysis and interpretation, journal writing. JF: design and facilitation of the studies, interpretation of results and statistics, design of figures and tables, proof-reading. PJ: design of the studies, interpretation of results and statistics, design of figures and tables, proof-reading.

### Conflict of Interest Statement

The authors declare that the research was conducted in the absence of any commercial or financial relationships that could be construed as a potential conflict of interest.
